# Use of Nonresponse Adjustment Factors for the Social Determinants and Health Equity Module in the Behavioral Risk Factor Surveillance System, 2022

**DOI:** 10.1016/j.focus.2025.100321

**Published:** 2025-02-06

**Authors:** Jason Hsia, Machell Town

**Affiliations:** 1 Division of Population Health, Centers for Disease Control and Prevention, Atlanta, Georgia

**Keywords:** Unit nonresponse, nonresponse adjustment, social determinants of health, social needs, Behavioral Risk Factor Surveillance System

## Abstract

**Introduction:**

The 2022 Behavioral Risk Factor Surveillance System introduced an optional module called social determinants and health equity to address health-related social needs, thereby supporting studies of social determinants of health. There were nonignorable nonresponses to the social determinants and health equity module.

**Methods:**

Nonresponse referred to an interviewee who completed the Behavioral Risk Factor Surveillance System core survey but chose not to answer any of the social determinants and health equity module questions. The study included 2022 Behavioral Risk Factor Surveillance System participants from 39 states; Washington, DC; and 2 territories. The sequential multiple imputation approach was used to impute the household income. Multivariable logistic regression was used to estimate the propensity of not answering any questions in social determinants and health equity. A nonresponse adjustment factor was developed for each state using a propensity score estimated from the logistic regression model.

**Results:**

Florida, California, and New Jersey had the highest nonresponse rates at 29.3%, 26.4%, and 25.6%, respectively. After excluding the outlier Puerto Rico, the median value of nonresponse adjustment factors for states ranged from 1.09 in Idaho to 1.67 in Florida.

**Conclusions:**

The nonresponse adjustment will mitigate the nonresponse bias in the analysis of social determinants and health equity data. The adjustment factor developed by the authors will be useful for analysts from various states, programs, and institutions studying social determinants and health equity.

## HIGHLIGHTS

• The nonresponse rates for the 2022 Behavioral Risk Factor Surveillance System social determinants and health equity (SDHE) module are high.
• Nonrespondents to the SDHE module have demographics different from those of respondents.
• We developed state-specific weight adjustment factors for nonresponses.
• This adjustment would help to mitigate nonresponse bias in SDHE data analysis.

## INTRODUCTION

The social determinants of health (SDOH) are 1 of the 3 main areas of focus for *Healthy People 2030*.^1^ In studying SDOH, researchers need to differentiate individual social needs from broader, population-based social determinants.^2^ The Behavioral Risk Factor Surveillance System (BRFSS), the world's largest state-based annual telephone-based health survey, collects data on participants’ health, use of preventive services, healthcare access, and health-related behavioral risk factors in 50 states, the District of Columbia, and participating U.S. territories.^3^ An optional module for BRFSS, social determinants and health equity (SDHE), was developed for 2022 to assess health-related social needs.^3^^,^^4^ The SDHE module, along with BRFSS as well as the Social Vulnerability Index, provides a data source for SDHE analysis, playing an important role for the Centers for Disease Control and Prevention. Thirty-nine states, the District of Columbia (DC), Puerto Rico, and the U.S. Virgin Islands (referred to as states), included the SDHE module in their 2022 state BRFSS surveys. According to its protocol, the BRFSS placed all state-selected optional modules after the core survey, which is the survey used by all states, DC, and participating territories. Almost 17% of the total who had the opportunity to respond to the optional SDHE module (56,197 respondents) took part only in the BRFSS core survey (*n*=338,778); they did not respond to any of the questions in the optional SDHE module. Studies using responses to the SDHE module produce inferential biases if analysts ignore this problem. The nonresponse was not dispersed proportionally among the various demographic groups.

The authors aimed to analyze the problem of the nonresponse not being distributed proportionally among the different demographic groups. The authors derive an adjustment factor for each respondent to mitigate the biases resulting from the unit's nonresponse to the SDHE module. The authors discuss how this change will benefit states, programs, and other organizations and affect the analysis of SDOH using data from the 2022 BRFSS SDHE module.

## METHODS

The BRFSS is an annual state-based telephone survey conducted in each state for non-institutionalized U.S. adults. All states, DC, and participating territories are asked the same essential questions (core survey), which address demographic information, health-related risk behaviors, chronic health issues, accessibility to health care, and use of preventive treatments. Only 39 states, DC, and 2 territories chose to incorporate the optional SDHE module into their BRFSS in 2022. The SDHE module, placed after the core survey, consists of 10 questions that assess health-related social needs^4^—(1) security-based needs, such as the extent of unemployment or underemployment, reliance on food assistance programs, limited food availability, inability to afford rent or utility payments, risk of utility service disconnection, and absence of transportation, and (2) social support needs, comprising overall life satisfaction, receiving social and emotional support, and feeling socially and emotionally isolated—and level of stress. Nonresponse refers to an interviewee who completed the core survey but chose not to answer any of the SDHE module questions.

Both unweighted and weighted nonresponse rates were estimated. The weighted nonresponse rates were estimated using the BRFSS analysis weight. The authors utilized the Rao–Scott chi-square test to examine any differences in distributions of demographic variables between respondents and nonrespondents. The authors employed multivariable logistic modeling to obtain a propensity score of unit response to SDHE for each state.^5^

$$Logit\;\left(y_{i}=1\right)=\sum w_{i}\beta_{ik},\;i=1\ldots 282,581;k=1\ldots K$$

In the logistic model, weight ($w_{i})\;$used was the BRFSS analytic weight, the dependent variable ($y_{i})\;$was the response status to the SDHE ($y_{i}=1$ for response and 0 for nonresponse), and K independent variables ($\beta_{ik})\;$included age categorized into 5-year group (18−24, 25−29, 30−34, 35−39, 40−44, 45−49, 50−54, 55−59, 60−64, 65−69, 70−74, 75−79, ≥80 years), sex, race and ethnicity (non-Hispanic White, non-Hispanic Black, American Indian or Alaskan Native, Asian, Native Hawaiian or other Pacific Islander, multiracial, Hispanic), employment status (employed for wages, self-employed, out of work for 1 year or more, out of work for <1 year, homemaker, student, retired unable to work), marital status (married, divorced, widowed, separated, never married, unmarried couple), home ownership (own, rent, other arrangement), education attainment (never attended school, Grade 1−8, Grade 9−11, Grade 12 or GED, college 1–3 years, college 4 year or more), health insurance coverage (yes, no), and household income (<$15,000; $15,000−$24,999; $25,000−$34,999; $35,000−$49,999; $50,000−$99,999; $100,000−$199,999; $200,000 or more), all of which were collected in the core survey. Next, the authors calculated the propensity score for the nonresponses using the estimated coefficients from the logistic model. This propensity score method is a more comprehensive version of weight adjustment (see pages 199–202 of Cox and Cohen^6^), assuming that missing data are random.^7^

Several independent variables included in the propensity score estimation (race and ethnicity, employment status, marital status, home ownership, education attainment, and health insurance coverage) had minor missing values. However, the BRFSS core survey showed that approximately 20% of the respondents had missing numbers for their household income. The authors used a multiple imputation method known as sequential regression imputation^8^ to fill in the missing variables. This method utilizes a sequential approach to impute missing values for several variables in a certain sequence. It also incorporates Bayesian techniques to modify the regression coefficients used for prediction and to incorporate residuals into the imputed values.^9^ The authors acquired 5 imputed datasets for every state. A commonly used method of combining separately imputed state data sets into one all-states data set is to stack imputed state data up in order, that is, stack all first imputed state data sets to make the first imputed combined data set, followed by stacking all second imputed state data sets to make the second combined data set, and so forth. Instead of using this commonly used approach, the authors selected the best state data from the 5 imputed data sets, that is, a specific imputed state data that were nearest to the income distribution of all 5 imputed data sets for each state through a goodness of fit test. The median (IQR) of *p*-values for all states was 90.1% (4.8%), showing a good fit. The authors then stacked the best state data set together to make the final complete data set for the propensity score calculation.

The primary adjusted weight (PAW) was calculated by multiplying the BRFSS analytic weight, used in the core survey, by the inverse of the propensity score. The PAWs were then trimmed at the maximum of BRFSS analytic weights, 54,390.5 or about 98th percentile of PAWs, to strike a balance between unbiasedness and precision. The recalibration of the trimmed weights was performed to align them with the overall state total as if they were estimated using only the core survey (see pages 204–205 of Cox and Cohen^6^). That is, after the trimming procedure, the authors multiplied all weights by a ratio to get the final adjusted weight. This ratio is calculated by dividing the total of all weights before the trimming by the sum of all weights after trimming. Finally, the final adjustment factor for overcoming the unit nonresponse problem was calculated as the ratio of the final adjusted weight over BRFSS analytic weight. All analyses were carried out using SAS survey procedures, Version 9.4 (SAS Institute).

## RESULTS

Forty-two states with 338,778 respondents to the core questions had the opportunity to respond to the SDHE module. A total of 282,581 adults provided responses to at least 1 question of the SDHE module. Data in Table 1 show that those who opted out of participating in the SDHE module had demographic distributions different from those who participated. These differences were in age, sex, race and ethnicity, employment status, marital status, home ownership, educational attainment, health insurance coverage, and household income. A higher percentage of nonrespondents were younger, male, non-Hispanic Black or Hispanic, without home ownership, less educated, lacking health insurance, and having lower household income than the respondents.

**Table 1:** Comparison of the Distribution of Demographic Characteristics Between Nonrespondents and Respondents to the Social Determinants and Health Equity Module, Behavioral Risk Factor Surveillance System, 39 States^a^, DC, and 2 Territories,^b^ 2022

Demographic characteristic	Sample size	Nonrespondent (%)^c^	Respondent (%)^c^	*p*-value^d^
Age, years	331,796			<0.001
18–34	55,679	36.69	27.71	
35–49	65,665	23.68	22.93	
50–64	87,654	21.37	24.95	
≥65	122,798	18.26	24.41	
Sex	338,778			<0.001
Male	159,120	50.39	48.25	
Female	179,658	49.61	51.75	
Race and ethnicity	328,070			<0.001
NH White	248,323	50.35	59.24	
NH Black	26,344	13.73	11.01	
AI/AN	4,835	1.45	1.26	
Asian	7,711	7.53	5.64	
Hispanic	34,060	23.05	19.45	
Other	6,797	3.90	3.39	
Employment status	330,152			<0.001
Employed	169,466	60.79	56.64	
Unemployed	12,474	5.78	4.99	
Retired	105,986	16.70	21.87	
Other	42,226	16.73	16.50	
Marital status	335,051			<0.001
Married	174,445	48.35	50.62	
Divorced/widowed/separated	87,415	18.16	20.56	
Never married/unmarried couple	73,191	33.49	28.82	
Home ownership	335,219			<0.001
Yes	239,972	60.95	69.91	
No	95,247	39.05	30.09	
Educational attainment	336,919			<0.001
Lower than high school	19,862	12.61	11.76	
High school	82,379	28.35	27.20	
Higher than high school	234,678	59.04	61.04	
Health insurance coverage	324,965			<0.001
Yes	307,016	88.68	91.74	
No	17,949	11.32	8.26	
Household income, $	264,854			0.005
<25,000	42,481	17.70	17.15	
25,000–49,999	67,623	26.96	25.40	
50,000–99,999	81,661	28.12	28.76	
100,000–199,999	55,403	19.70	21.06	
≥200,000	17,686	7.51	7.64	

a Alabama, Alaska, Arizona, California, Connecticut, Delaware, Florida, Georgia, Idaho, Indiana, Iowa, Kansas, Kentucky, Maine, Maryland, Massachusetts, Michigan, Minnesota, Mississippi, Missouri, Montana, Nebraska, Nevada, New Hampshire, New Jersey, New Mexico, North Carolina, Ohio, Oklahoma, Rhode Island, South Carolina, Tennessee, Texas, Utah, Vermont, Washington, West Virginia, Wisconsin, and Wyoming.

b Puerto Rico and U.S. Virgin Islands.

c BRFSS analytic weight applied.

d *p*-value from the Rao–Scott chi-square test. AI/AN, American Indian/Alaska Native; BRFSS, Behavioral Risk Factor Surveillance System; DC, District of Columbia; NH, non-Hispanic.

The median of unweighted nonresponse rate among all states that included the SDHE module was 15.1%. More than one third of the states had a nonresponse rate over 19%. Florida (29.3%), California (26.4%), and New Jersey (25.6%) had the highest unweighted nonresponse rates. The weighted (BRFSS analytic weight) nonresponse rates exceeded the unweighted nonresponse rate for each state. The nonresponse adjustment factor distribution by state is presented in Table 2. After excluding the outlier Puerto Rico, which had the lowest nonresponse rate (2.6%), the median value of nonresponse adjustment factors for states ranged from 1.09 in Idaho to 1.67 in Florida.

**Table 2:** Nonresponse Rate and Distribution of Nonresponse Adjustment Factors by State, Behavioral Risk Factor Surveillance System, 39 States^a^, DC, and 2 Territories,^b^ 2022

		Nonresponse rate (%)		Adjustment factor		
State^c^	*n*	Unweighted	Weighted^d^	Q1	Median	Q3
Florida	13,393	29.3	41.8	1.487	1.668	2.012
California	10,952	26.4	25.9	1.263	1.336	1.438
New Jersey	8,209	25.6	25.7	1.254	1.322	1.425
Nevada	3,188	23.5	26.9	1.265	1.346	1.460
Georgia	9,236	22.7	28.0	1.270	1.360	1.507
Texas	14,245	21.9	29.9	1.303	1.400	1.543
Ohio	10,784	21.5	22.3	1.221	1.279	1.344
North Carolina	4,505	20.7	21.5	1.158	1.221	1.356
DC	3,237	20.1	25.6	1.192	1.289	1.476
South Carolina	10,037	19.9	22.3	1.220	1.274	1.342
Tennessee	5,266	19.7	23.2	1.183	1.269	1.401
Indiana	10,466	19.1	21.1	1.208	1.253	1.324
Rhode Island	5,893	19.0	19.9	1.182	1.231	1.305
Connecticut	9,784	18.6	19.1	1.172	1.227	1.296
Arizona	10,185	18.6	23.8	1.248	1.298	1.371
Maryland	10,636	18.5	19.9	1.176	1.235	1.316
Wisconsin	11,276	18.0	19.8	1.188	1.229	1.290
Delaware	3,987	17.9	18.7	1.171	1.215	1.277
Minnesota	16,821	15.7	16.4	1.161	1.185	1.217
New Hampshire	6,757	15.6	18.5	1.162	1.217	1.278
Michigan	6,468	15.1	17.0	1.142	1.188	1.259
Wyoming	4,142	14.8	17.0	1.143	1.177	1.240
Iowa	8,949	14.5	16.3	1.144	1.176	1.232
Maine	10,646	14.5	16.0	1.147	1.177	1.218
Vermont	8,811	14.2	15.6	1.147	1.178	1.216
Utah	9,826	14.1	15.2	1.135	1.163	1.208
Kansas	11,247	12.9	15.8	1.131	1.169	1.228
New Mexico	4,758	12.8	15.3	1.123	1.165	1.215
Alabama	4,506	12.5	15.2	1.098	1.141	1.215
Alaska	5,865	12.4	13.5	1.106	1.143	1.196
Massachusetts	11,029	12.4	12.2	1.092	1.128	1.175
Washington	26,152	12.2	14.2	1.115	1.151	1.207
Oklahoma	2,796	11.4	12.3	1.084	1.124	1.184
U.S. Virgin Islands	1,531	11.4	14.2	1.037	1.108	1.211
Mississippi	4,239	11.0	12.8	1.086	1.124	1.180
Kentucky	4,023	10.7	15.3	1.085	1.131	1.230
West Virginia	4,981	9.7	11.7	1.089	1.117	1.163
Missouri	7,438	9.5	11.8	1.083	1.111	1.165
Nebraska	3,677	9.2	9.2	1.070	1.095	1.124
Idaho	6,280	8.8	9.0	1.071	1.087	1.114
Montana	7,048	7.8	9.5	1.070	1.093	1.122
Puerto Rico	5,509	2.6	2.9	1.014	1.022	1.035

a Alabama, Alaska, Arizona, California, Connecticut, Delaware, Florida, Georgia, Idaho, Indiana, Iowa, Kansas, Kentucky, Maine, Maryland, Massachusetts, Michigan, Minnesota, Mississippi, Missouri, Montana, Nebraska, Nevada, New Hampshire, New Jersey, New Mexico, North Carolina, Ohio, Oklahoma, Rhode Island, South Carolina, Tennessee, Texas, Utah, Vermont, Washington, West Virginia, Wisconsin, and Wyoming.

b Puerto Rico and U.S. Virgin Islands.

c States are sorted in descending order by unweighted nonresponse rate.

d BRFSS analytic weight was applied. BRFSS, Behavioral Risk Factor Surveillance System; DC, District of Columbia.

Table 3 displays the estimated total and proportion of 10 variables pertaining to security-based needs, social support needs, and stress levels before and after applying the nonresponse adjustment. After applying the nonresponse adjustment, the total number of eligible adults from all 42 states represented by the 282,581 responders to the SDHE was 205,340,628. Conversely, the weighted total amounted to only 158,110,101 without implementing the adjustment. The underestimation was the same for all 10 variables (Table 3). For example, of the 42 states, a total of 25.5 million individuals relied on food assistance programs. However, without accounting for nonresponse adjustment, the number would be fewer than 20 million. In addition, after applying the nonresponse adjustment, the estimated proportions of life dissatisfaction, lack of social and emotional support, perceived social isolation, stress, unemployment or reduced employment, reliance on food assistance programs, limited food availability, inability to afford rent or utilities, experienced threat of utility service disconnection, and lack of transportation were higher than the proportions estimated without the adjustment. Furthermore, the SEs of the proportions were marginally higher when the adjustment was applied than when it was not applied.

**Table 3:** Estimated Totals and Proportions of Variables in the Social Determinants and Health Equity Module Without and With Nonresponse Adjustment, Behavioral Risk Factor Surveillance System, 2022, 39 States^a^, DC, and 2 Territories,^b^ 2022

SDHE item	*n*	Estimates without adjustment^c^				Estimates with adjustment^c^			
		T^d^	se(T)	P(%)^e^	se(P)	T^d^	se(T)	P(%)^e^	se(*p*)
Total		282,581	158.1	0.4			205.3	0.6	
Overall life satisfaction									
Dissatisfied^f^	15,478	9.8	0.2	6.3	0.11	13.0	0.2	6.4	0.12
Satisfied^g^	264,212	146.5	0.4	93.7	0.11	190.0	0.6	93.6	0.12
Total		279,690	156.3	0.4	100.0		202.9	0.6	100.0
Receiving social and emotional support									
Rarely/never/sometimes	60,032	38.8	0.3	25.0	0.19	51.4	0.5	25.5	0.21
Usually/always	217,908	116.5	0.4	75.0	0.19	150.3	0.6	74.5	0.21
Total		277,940	155.4	0.4	100.0		201.8	0.6	100.0
Feeling socially isolated									
Always/usually/sometimes	82,816	49.8	0.3	31.9	0.19	65.3	0.5	32.3	0.21
Rarely/never	196,395	106.1	0.4	68.1	0.19	137.1	0.6	67.7	0.21
Total		279,211	155.9	0.4	100.0		202.4	0.6	100.0
Level of stress within the last 30 days									
Always/usually/sometimes	94,640	59.5	0.4	38.5	0.20	77.9	0.5	38.8	0.22
Rarely/never	182,771	95.0	0.4	61.5	0.20	122.7	0.6	61.2	0.22
Total		277,411	154.5	0.4	100.0		200.6	0.6	100.0
Loss or reduced employment^h^									
Yes	25,703	19.9	0.3	12.8	0.16	26.9	0.4	13.3	0.17
No	253,551	135.9	0.4	87.2	0.16	175.4	0.6	86.7	0.17
Total	279,254	155.8	0.4	100.0		202.3	0.6	100.0	
Reliance on food assistance programs^h^									
Yes	28,255	19.3	0.2	12.4	0.15	25.5	0.3	12.6	0.16
No	251,886	136.9	0.4	87.6	0.15	177.3	0.6	87.4	0.16
Total	280,141	156.2	0.4	100.0		202.8	0.6	100.0	
Limited food availability^h^									
Always/usually/sometimes	29,619	21.9	0.3	14.1	0.16	29.3	0.4	14.5	0.17
Rarely/never	249,564	133.8	0.4	85.9	0.16	172.9	0.6	85.5	0.17
Total		279,183	155.7	0.4	100.0		202.1	0.6	100.0
Inability to afford mortgage/rent/utility									
Payments^h^									
Yes	25,201	18.6	0.2	12.0	0.14	24.9	0.3	12.3	0.16
No	253,878	136.8	0.4	88.0	0.14	176.8	0.6	87.7	0.16
Total	279,079	155.4	0.4	100.0		201.7	0.6	100.0	
Experienced threat of utility service disconnection^h^									
Yes	16,814	11.8	0.2	7.6	0.12	15.6	0.3	7.7	0.13
No	262,330	143.7	0.4	92.4	0.12	186.3	0.6	92.3	0.13
Total	279,144	155.5	0.4	100.0		201.8	0.6	100.0	
Absence of transportation^h^									
Yes	18,181	12.8	0.2	8.3	0.12	17.1	0.3	8.5	0.14
No	260,754	142.5	0.4	91.7	0.12	184.5	0.6	91.5	0.14
Total	278,935	155.3	0.4	100.0		201.6	0.6	100.0	

a Alabama, Alaska, Arizona, California, Connecticut, Delaware, Florida, Georgia, Idaho, Indiana, Iowa, Kansas, Kentucky, Maine, Maryland, Massachusetts, Michigan, Minnesota, Mississippi, Missouri, Montana, Nebraska, Nevada, New Hampshire, New Jersey, New Mexico, North Carolina, Ohio, Oklahoma, Rhode Island, South Carolina, Tennessee, Texas, Utah, Vermont, Washington, West Virginia, Wisconsin, and Wyoming.

b Puerto Rico and U.S. Virgin Islands.

c Adjustment of unit nonresponse to SDHE.

d The total number estimated in millions.

e Proportion.

f Include very dissatisfied.

g Include very satisfied.

h During the last 12 months. DC, District of Columbia; SDHE, Social Determinants and Health Equity.

## DISCUSSION

The BRFSS is a comprehensive surveillance system that monitors behavioral risk factors for the prevention of chronic diseases. The data collection mode is telephone, encompassing both landlines and cellular phones. Consistent growth in the breakoff rate in the core survey of BRFSS, the comprehensive surveillance system that monitors behavioral risk factors for the prevention of chronic diseases, was noted by Hsia et al.^10^ as occurring despite enhanced data collection protocols and diligent field operations. Furthermore, a significant number of nonrespondents to the optional modules that are included after the core survey is inevitable. Nonresponse to the SDHE is a serious problem if respondents are not representative of all respondents to the core survey. Indeed, Rao-Scott chi–square tests revealed a significant difference in the distribution of demographic characteristics between individuals who did and those who did not respond to the SDHE module because nonrespondents were more likely younger, male, non-Hispanic Black, or Hispanic and had a lower SES. Disregarding the nonresponses as if the missing data were completely random introduces bias into the inference. The adjustment factor this study provides accounts for unit nonresponse in the SDHE module. The new analysis weight, derived from the adjustment factor and the BRFSS analysis weight, is utilized to analyze SDHE data and mitigate nonresponse bias.

The bias from estimating the population total is straightforward and easily understood. Table 3 shows a significant decrease in the total number of undesirable outcomes such as individuals requiring social support or lacking security-based needs when the nonresponses were disregarded compared with when the nonresponses were addressed. Quantifying the missing population becomes challenging when the unit nonresponse problem is disregarded, making it difficult to confine the problem within limits.

Estimates were made for both unweighted and weighted nonresponse rates. The unweighted nonresponse rate is the proportion of persons who opted not to provide answers to any questions in the SDHE module. The weighted nonresponse rate represents the estimated proportion of individuals in the target population who did not participate in the SDHE survey. The observation that the weighted values exceeded the unweighted values for all states provides an alternative perspective on the uneven distribution of demographic attributes among nonrespondents.

The nonresponse adjustment employed in this study is implemented using a propensity score approach. Although the assumption of this approach is still that responses are missing at random, it is a more comprehensive approach than weighting class calibration, analogous to multivariable logistic regression and crosstabulation analysis. The propensity score method allows for the inclusion of a greater number of factors that affect nonresponse than the weighting class approach. As a result of applying nonresponse adjustment, all adjustment factors exceeded 1, meaning that the final adjusted analytic weight would be greater than the original BRFSS analytic weight. Therefore, the variance obtained using the final adjusted analytic weight would be higher than that obtained using the original BRFSS analytic weight. This is primarily attributed to a reduced sample size after nonresponse and variation among demographic characteristics considered. The higher the nonresponse rate, the greater nonresponse adjustment factor.

Nonresponse adjustment factors were calculated for each state to analyze the 2022 SDHE module data. Using the adjustment factor or applying the final analytic weight to each eligible observation are a viable option. Users can contact the authors for any technical inquiries regarding the adjustment factor for nonresponse to SDHE. The SDOH is a top concern for the Centers for Disease Control and Prevention. As part of its assistance to study the SDOH, BRFSS incorporated the SDHE optional module. To address the problem of nonresponse in studying SDHE, the nonresponse adjustment factor to reduce the nonresponse bias in the analysis can be used. This adjustment factor will be directly useful for all analysts of 2022 SDHE data from various states, programs, and institutions. In addition, the methodology the authors proposed in this paper can be used to handle similar situations, including nonignorable nonresponse to other optional modules or some questions of BRFSS.
